# Supplementary material for: Patterns of Intron Gain and Loss in Fungi
Source: PLoS Biol. 2004 Nov 30;2(12):e422. doi: 10.1371/journal.pbio.0020422 (PMC532390; doi:10.1371/journal.pbio.0020422)
Supplement: Table S1 — Also available at http://genes.mit.edu/NielsenEtAl/. (4.3 MB ZIP). [file pbio.0020422.st001.zip › NielsenEtAl/html/267.html]

AN5535.1.NCU04505.1.MG00102.1.FG03976.1


```
 CLUSTAL W (1.82) Multiple Sequence Alignments - Introns Inserted


Sequence 1: NCU04505.1	757 aa
Sequence 2: MG00102.1	728 aa
Sequence 3: FG03976.1	689 aa
Sequence 4: AN5535.1	685 aa
Alignment Length: 775 aa
Number Identitical Residues: 276 aa
Alignment Score (without introns) 15543


MG00102.1 	MDGLDDFEKQLAADKAEREHEQERAER-KEKRRHHHR-RDRSSDRTGDRE--RHRDHDRD
NCU04505.1	MDSLEDFEKQLAAEREERERAKEKEQRRKHKHHHHHR-RDRSSDRDRDRAGNRDKDTERD
FG03976.1 	MDGLDEFEKTLAAEKAERERAERHDDEKRHRKHRHHH-RHDS-----------ERDGEHD
AN5535.1  	-MTLEDFEKSLAEER-ERERRREKSDGDKHRHRHHDRERDRDRDRDHRRDRDRSREHRHR
          	   *::*** ** :: ***: ..: :  :.::::*.:.*. . .       .  ::  : 

MG00102.1 	SDRHR----HKHRRHDSRE-RERDRDEHGHRHKRSRKDEDENDRDKDSSRRRHRHRNGSP
NCU04505.1	KDRHRDRDSDRHRSHRSRDDHDEEDDKDGHRHKRSRHSR-HDDHHEEESRHRHRHRDRSG
FG03976.1 	KDRHR----HRHRRR-----HDEEDD--GHRSKRSRHSR---DDDDGGHRSRHRHSD---
AN5535.1  	HRSHR---HHSRSRERGSDRNNESHRDDGHRHKRSRQSS---DHGEDRDHHKRRHRE---
          	   **    . :  . . . .:..  . *** ****:.    *  .   : ::** :   

MG00102.1 	SRRNGREVNE-------SGEPSYIQAHDPKEDLPLPDEE-TPSAEAAAR---PVRDSWMT
NCU04505.1	DRYSERDREHRSDRRRSTKDTKDLKPTDAKEDLPLPDEEKAPMDVDKPA---LVRDSWMT
FG03976.1 	DRYR--------------------KTRDAKEDLPIPDEEQPQSQDTIDRSKSSQRDAWMT
AN5535.1  	--------------------------KEGREDEPATTAEITQEEPARLK-----RDAWME
          	                           : :** * .  * .             **:** 

MG00102.1 	APSALDVDYVHRSQKD------KGPAKKEPEQRVISERELNSGVLAAMNSGKAFED----
NCU04505.1	APSAIDVDYVQRGRKR------SPSPKKEEPKRVLHAREVNRGLAEMENWSMPVERGVER
FG03976.1 	APSSIEVDYIHRPDKN------KSPPPKEEPKRVVSKREINNHIQDIND-GKSLDE----
AN5535.1  	APSALDIDYVQRREKTRLEQEPKPRMLQADFELKIHNKELNTHLRDLRD-GKALEEIE--
          	***::::**::*  *   .....    :   :  :  :*:*  :    : . ..:     

MG00102.1 	-LQVPTQTEVSYKFGDEGSQWRMTKLKGVYDTAKRTGRSVDEIGAETFGSIREFDDAREE
NCU04505.1	GQEPPRKREVNYTFGDEGSQWRMTKLKAVYTTAEQTGKTVEEVALERFGSLEEFDEAREE
FG03976.1 	-LDIPSQPTVDYTFGDAGSSWRMTKLKAVYTAAEESGKPVEEVAMERFGDLRHFDDAREE
AN5535.1  	--QEPAEHEVNYTFGDAGSSWRMTKLKGVYREAKESGRSEEEVAIERFGDLRSFDDAREE
          	  : * :  *.*.*** **.*******.**  *:.:*:. :*:. * **.:. **:****

MG00102.1 	RIEMDRRRTYGSGYKGKEVPTGELYKER--NIDDSRPSEVQDDAESPPEQGIVVDHPP--
NCU04505.1	KIEVDRRKLYGEGYAGKEKPTGELYEERVAKMRSQRPESPDREESSPPAQGVIIEDQLQK
FG03976.1 	KEEMERRRIYGKDYKGHDKPTGDLYRER---IKNQHKDSPPPE----PEQGTIVPDAHPQ
AN5535.1  	EAELERRETYGEGYVGKEKPTGELFRER-------------------KQQENVHRDVH--
          	. *::**. **..* *:: ***:*:.**                     *  :  .    

MG00102.1 	---AARIDQTTLNRMRA~AMMNAKLRRAPDTEKLEAEYNAAMASFSSG------VSNDRA
NCU04505.1	---APPIDQSTLNRMRA~ALMKAKLRKAPEAAKLEEEYNAAMAAFQAGNAFQAGSVSGAA
FG03976.1 	TTTTPVMDQTSLNRLRA~QMMKAKLRKAPNAAQLEEEYNEAAAMFSAAQ------SNPEA
AN5535.1  	----EHLRDPEKEFSAP1QMMRAKLRAAPDAAELEEQYNAAAAAMANRK-------EPDV
          	      : :.  :   .  :*.**** **:: :** :** * * :   .       .  .

MG00102.1 	VVLDASHSRMLAAGGQRAETKALD-TKRGRERGTLVANDDMTIDDMVREEKRTRGQAGGD
NCU04505.1	VVLDASHNRMLAG--PRGEVVAVTNTKRGRERGTVVENEDMSIDDMVREERRTRGQAGGE
FG03976.1 	VVLGVMDNRQLAG--RRNEAKVIT-NKRGRERGNVEENTEMTIDDMVREERRTKGQAGGE
AN5535.1  	MVLGVMENRMLAG--KRNEVKAIQ-TKRGLERGKVEENEDMTIEDMVQEERRSRGQFGGE
          	:**.. ..* **.   * *. .:  .*** ***.:  * :*:*:***:**:*::** **:

MG00102.1 	GMKMAERIARDGKFD~NDLEYMEENAERLAKRTHKSDAALKALAVGEFQRMNKILDSCPL
NCU04505.1	GLRLAERIAKDAKFD~DDLEYLDENAAKLAKRVHKNEASLKNVAVAEYRKLNRILDTCPL
FG03976.1 	GLRLAERIAKDGKFD~NDLDYMDENAEKLAKRLHKSDINLKNMAVSEFQKMSRALDNCPL
AN5535.1  	GRRLAERIAKDSRFE0NDLEYMDDNASKLARRVHRSEIDLRNTTINELQKMNKILDNCPL
          	* ::*****:*.:*: :**:*:::** :**:* *:.:  *:  :: * :::.: **.***

MG00102.1 	CHHEDREPPRNLPIAPVISLATRVYMTLAPGPELNNGAEGGAVLVPLSHRQNLLECDDDE
NCU04505.1	CHHEEQVPPKNLPVAPIISLATRVYLTLPTSPELT-GAEGGALIVPISHRTNLLECDDDE
FG03976.1 	CHHEDKNQP---PLAPVIALGTRAFLTIATEPEIS---SGGAVIAPLAHRSNLLECDDDE
AN5535.1  	CHHEDTNTP---PVAPVVSLATRVYLTLPTEPEIS---PGSATIVPIQHRTNLLECDDDE
          	****:   *   *:**:::*.**.::*:.. **:.    *.* :.*: ** *********

MG00102.1 	WEEMRNFMKSLTRLYHDQGREVLFYENAAAPNRRGHAHMVAVPIPYEQGDTAPAFFKEAM
NCU04505.1	WEEIRNFQKSLTRLYHEQGREVLFYENAAAPGRRLHAAMMAVPIPWDLGDTAPAFFREAM
FG03976.1 	WEEIRNFMKSLTRMYHEQGREVVFYENAAQPHRRMHAAMVAVPIPYEEGAMAPAYFKEAF
AN5535.1  	WEEIRNFMKSLTRMYHDQGRDVIFYENAAAPHRKRHASMEAVPLPYSLGETSPAFFKEAI
          	***:*** *****:**:***:*:****** * *: ** * ***:*:. *  :**:*:**:

MG00102.1 	LTVGEEWSQHKKYVDTAKKAREG-LGKLAFRRSIAKEAPYFHVWFNLDGGLGHVVEDESS
NCU04505.1	LSADDEWAQHKKVIDTGKAAREGGMGKLAFRRSIAKEMPYFHVWFNLDGGLGHVVENSER
FG03976.1 	LSADEEWSQHRKIIDTGAKARDG-MGRSAFRRSIAKEMPYFHVWFTLDGGLGHVVEDAGR
AN5535.1  	LSSDEEWTQHRKLIDTLTKSKQG-LGRNAFRRSLAKEMPYFHVWFQLDGGLGHIVEDANR
          	*: .:**:**:* :**   :::* :*: *****:*** ******* *******:**:   

MG00102.1 	WPRGDQFAREIIGGMLDSDMDLIKKQPRWSRS-DSRADDFKKRWRKFDWTRVLTEGQ
NCU04505.1	WPRGDLFAREIIGGMLDSEPDVIKRQGRWSRS-DERVDGFKKRWRKFDWTRVLTEGA
FG03976.1 	WPKGDLFAREVLGGIVDAEPHIIKRQGKWTRG-DPRVEGFKKGWRKFDWTRMLAEG-
AN5535.1  	WPKGDLFAREVIGGMLDVGPEVIKKQGRWTRGGDRRVGGFQSRWRKFDWTRVLVEG-
          	**:** ****::**::*   .:**:* :*:*..* *. .*:. ********:*.**
```
